# Histones from Avian Erythrocytes Exhibit Antibiofilm activity against methicillin-sensitive and methicillin-resistant *Staphylococcus aureus*

**DOI:** 10.1038/srep45980

**Published:** 2017-04-05

**Authors:** Megan Rose-Martel, Garima Kulshreshtha, Nahom Ahferom Berhane, Joelle Jodoin, Maxwell T. Hincke

**Affiliations:** 1 Department of Cellular and Molecular Medicine, Faculty of Medicine, University of Ottawa, Ontario, K1H 8M5 Canada; 2 Department of Innovation in Medical Education, Faculty of Medicine, University of Ottawa, Ontario, K1H 8M5 Canada

## Abstract

*Staphylococcus aureus*, a human pathogen associated with many illnesses and post-surgical infections, can resist treatment due to the emergence of antibiotic-resistant strains and through biofilm formation. The current treatments for chronic biofilm infections are antibiotics and/or surgical removal of the contaminated medical device. Due to higher morbidity and mortality rates associated with overuse/misuse of antibiotics, alternate treatments are essential. This study reports the antibiofilm activity of avian erythrocyte histones against methicillin-sensitive *Staphylococcus aureus* (MSSA) and methicillin-resistant *Staphylococcus aureus* (MRSA). Fluorescence and scanning electron microscopy revealed membrane damage to bacteria in histone-treated biofilms. Histones and indolicidin (positive control) increased the expression of *apsS* and *apsR*, which are associated with the Antimicrobial Peptide (AMP) sensor/regulator system in *S. aureus*. The expression of *dltB*, and *vraF*, associated with AMP resistance mechanisms, were under histone inducible control in the biofilm-embedded bacterial cells. The time kill kinetics for histones against *S. aureus* revealed a rapid biocidal activity (<5 min). Purified erythrocyte-specific histone H5 possessed 3–4 fold enhanced antimicrobial activity against planktonic cells compared to the histone mixture (H1, H2A, H2B, H3, H4, H5). These results demonstrate the promise of histones and histone-like derivatives as novel antibiotics against pathogens in their planktonic and biofilm forms.

*Staphylococcus aureus* is a major human pathogen associated with a range of illnesses, from minor skin infections to life-threatening diseases, such as pneumonia, meningitis, endocarditis, toxic shock syndrome (TSS), bacteremia, and sepsis^1^. Moreover, *S. aureus* is one of the most common causes of postsurgical wound infections^2^. Among the species belonging to the *Staphylococcus* genus, *S. aureus* exhibits the highest prevalence (35.5%) for implant infections^3^. Generally, antibiotic treatment and surgical removal of the medical device becomes necessary to prevent chronic biofilm infection^4^. Unfortunately, the widespread use of antibiotics has selected for methicillin-resistant *Staphylococcus aureus* (MRSA). Compared to patients infected with methicillin-sensitive *Staphylococcus aureus* (MSSA), MRSA is associated with higher morbidity and mortality rates, as well as longer hospital stays^5^. In fact, Canadian hospitals reported a 17-fold increase in MRSA infections and colonization incidences from 1995 to 2007^6^. Furthermore, the American Center for Disease Control estimated >80,400 invasive infections and almost 11,300 deaths involving MRSA in the USA in 2011^7^.

Cationic antimicrobial peptides (CAMPs), such as cathelicidins, magainins and dermaseptins, are crucial members of the innate immune system. They possess hydrophobic residues, have an overall positive charge and can form amphipathic α-helical structures, ß-sheet structures or remain in a linear arrangement in a membrane-like milieu^8^. Although CAMPs may have both intracellular and extracellular targets, antimicrobial peptides mainly bind to the anionic constituents of the bacterial cell membrane, which pathogens cannot easily mutate. This enables CAMPs to avoid the common resistance mechanisms observed for classical antibiotics^9^. However, in response to CAMPs, many pathogens upregulate resistance mechanisms to improve their survival^10^. This reaction is mediated by activation of the three component sensor/regulator system *aps* (Antimicrobial Peptide Sensor), which alters the overall negative charge of the bacterial cell surface by i) D-alanylation of teichoic acids by the *dlt* operon, and/or ii) lysylination of phospholipids by MprF protein that decreases the attraction and binding of CAMPs. Another *aps* regulated gene locus, *vraFG*, is similar to the ABC transporter system; it confers resistance to CAMPs by either exporting the CAMPs from the bacterial cell or importing them for proteolytic inactivation^11^.

Histones, commonly known for their role in DNA stabilization and packaging, were first shown to possess antimicrobial activity in samples isolated from calf thymus^12^. Since then, histones have been identified in a variety of species as potent antimicrobial agents possessing all of the characteristics of CAMPs^13^. Histones are also the most abundant proteins associated with Neutrophil Extracellular Traps (NETs) - key players in the innate immune response in a variety of vertebrate species^14^. NETosis, the process that forms NETs, occurs when neutrophils release their nuclear content in order to eliminate pathogens extracellularly and is induced by several factors including *S. aureus* infections^15^,^16^.

Avian blood contains nucleated erythrocytes with DNA packaged by histones. It is therefore a resource for facile purification of histones, including the erythrocyte-specific H5^17^, which possesses 38% similarity to histone H1 and does not have a mammalian analog. Histone H5 is 190 amino acids in length, with a hydrophobic ratio of 28%, a total net charge of +61 and an isoelectric point of +12. To our knowledge the antimicrobial properties of histone H5 have not been previously studied. We have previously demonstrated that chicken erythrocyte histones exhibit antimicrobial activity towards a variety of Gram-positive and Gram-negative planktonic bacteria, and bind to bacterial cell wall components such as lipopolysaccharide (LPS) and lipoteichoic acid (LTA)^18^. Furthermore, mammalian red blood cell membranes were stable to chicken histones treatment and showed no hemolysis even at the highest concentration tested^18^.

In this study, we compare the antimicrobial and antibiofilm activity of histones extracted from chicken erythrocytes against MSSA and MRSA, and demonstrate antimicrobial protection against both biofilm forms. To explore the mechanisms underlying the antimicrobial activity, we investigated bacterial membrane damage using fluorescence approaches and scanning electron microscopy, and assessed upregulation of resistance genes. Furthermore, the relatively uncharacterized erythrocyte-specific linker histone H5 was purified and characterized as an antimicrobial peptide. We anticipate that these studies will lead to identification and development of novel histone-derived antimicrobial peptides.

## Results

### Assessment of Purified Histones and Histone H5 by Proteomics

**Figure 1:**
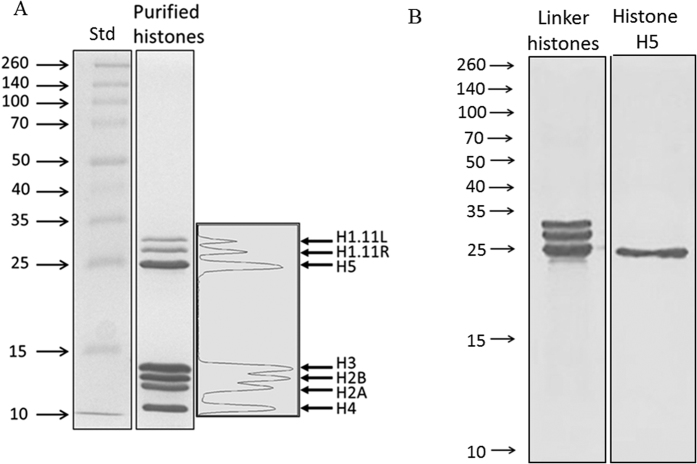
SDS-PAGE analysis of the TCA-precipitated histone mixture purified from chicken erythrocytes. (**A**) 15% acrylamide gel revealing 7 distinct bands which were analyzed using densitometry, and then excised for proteomics LC/MS/MS analysis. All core and linker histones were present: H1.11L, H1.11R, H2A, H2B, H3, H4 and H5 (see Table 1 for proteomics and densitometry results). (**B**) Linker histones and histone H5 separated by SDS-PAGE on a 15% gel. Lane (1) linker histones; lane (2) purified histone H5.

Histones were extracted and purified from chicken erythrocytes by sulfuric acid extraction and trichloroacetic acid (TCA) precipitation. This method yielded 2.3 ± 0.6 mg of histones/ml whole blood. SDS-PAGE analysis revealed 7 distinct bands which were analyzed by densitometry and subjected to proteomics analysis (Fig. 1A). The bands were identified as H1 (9.7% ± 1), H2A (15% ± 1), H2B (19% ± 1), H3 (24% ± 1), H4 (14.6% ± 0.4) and H5 (18% ± 1) (Table 1 and Fig. 1). Hence, the three most abundant proteins of the histone mixture are H2B, H3 and H5. The H1 bands were composed mainly of both H1.11L and H1.11R; there is a 93% identity between the histone H1 variants, with the H1.11R sequence being 6 amino acids shorter. Every histone possessed an exponentially modified protein abundance index (emPAI) > 700. Minor contaminants included SAP domain containing ribonucleoprotein (ACCN: Q5ZLP5), 60S ribosomal protein L7a (ACCN: F1NHA8), THO complex subunit 4 (ACCN: E1C2L5), peptidyl-prolyl cis-trans isomerase (ACCN: Q90ZK7), high mobility group proteins (ACCN: Q9YH06 and P40618), adenylate kinase 2 (ACCN: F1NJ73), myosin light polypeptide 6 (ACCN: P02607), small nuclear ribonucleoprotein Sm D3 (ACCN: Q5ZL58), hemoglobin subunits α-A and α-D (ACCN: F1NEY9 and P02001), each with emPAI values ranging from 1–5. Using spectral counting, we determined that these contaminants corresponded to <5% of the histone mixture in total.

Linker histones (histone H1 and H5) were extracted from chicken erythrocytes by perchloric acid extraction and TCA precipitation. Histone H5 was purified by ion exchange chromatography using a step salt gradient (Fig. 1B; Supplemental file, materials and methods). The purified histone H5 (densitometry > 98.9%) was determined by proteomics analysis to consist of 96.8% histone H5, with negligible contamination by histone H1 variants (H1.11R, H1.11L, H1.01, H1.03).

### Time Kill Kinetics

**Figure 2:**
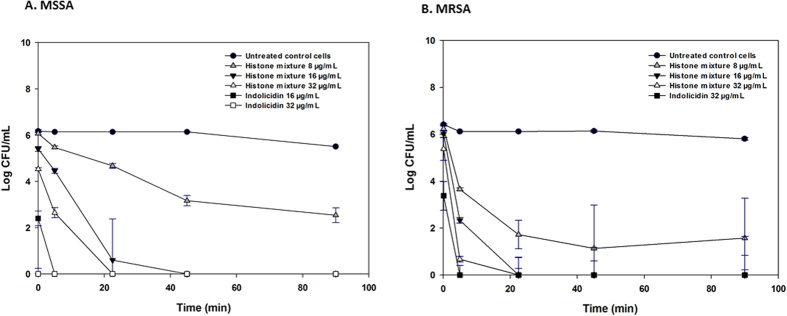
Killing kinetics for MSSA and MRSA following treatment with CAMPs. Growth kinetics were monitored for (**A**) MSSA and (**B**) MRSA following exposure to histones, indolicidin (positive control) at concentrations ranging from MIC to 4 × MIC for 0–90 min. Sterile water, pH 7.4 was used as a negative control. The nominal time point 0 was estimated to be 15 s, corresponding to the minimum time required to mix and pipet the bacterial cultures with their treatments into the 96 well microplate. Values represent the mean ± SD of n = 6 replicates at each time point.

To evaluate the biocidal activity of histones against MSSA and MRSA, a kill kinetics assessment was performed. The minimum inhibitory concentration (MIC) values of histones against MSSA and MRSA were 6 ± 1 μg/ml and 8 ± 2 μg/ml, respectively^18^. Histones showed a time dependent bactericidal activity, resulting in a 0.5 to 3.5 log_10_ reduction in viable MSSA within 5 min of exposure of histones (P ≤ 0.001; Fig. 2A). Within 22.5 min, a pronounced growth reduction (5.5 to 6 log_10_ reduction, P ≤ 0.001) was observed in the MSSA cells treated with histones. Bacterial growth was completely inhibited within a 45 min exposure to histones at 2 × MIC (16 μg/ml) and 4 × MIC (32 μg/ml). Wells containing bacteria treated with 2 x MIC and 4 × MIC concentrations of histones showed no sign of growth during 16 h of subsequent monitoring even for the 180 min treated samples (data not shown). The histones had a greater inhibitory effect against MRSA at all concentrations (1 × MIC, 8 μg/ml to 4 × MIC, 32 μg/ml) (Fig. 2B). A 2.5 to 5.5 log_10_ reduction in MRSA counts was observed within 5 min of exposure to the histones. Compared to the untreated control cells, a 3.5 log_10_ reduction was observed in MRSA after 22.5 min exposure to 1 x MIC of histones. At 2 x MIC and 4 x MIC of histones, complete MRSA growth inhibition was observed for samples treated for 22.5 min (Fig. 2B). After treatment for 180 min, there was no growth detected (>6 log reduction) (data not shown). Indolicidin was used as a positive control since it is known to be active against *S. aureus*^19^. The MIC of indolicidin was determined to be 16 μg/ml (MSSA) and 32 μg/ml (MRSA) (data not shown). Compared to untreated control cells, the exposure of indolicidin (16–32 μg/ml) resulted in a significant growth reduction (5.5 to 6 log_10_ reduction, P* ≤ *0.001) for both MSSA and MRSA planktonic cells within 5 min (Fig. 2A and B).

### Anti-Biofilm activity

**Figure 3:**
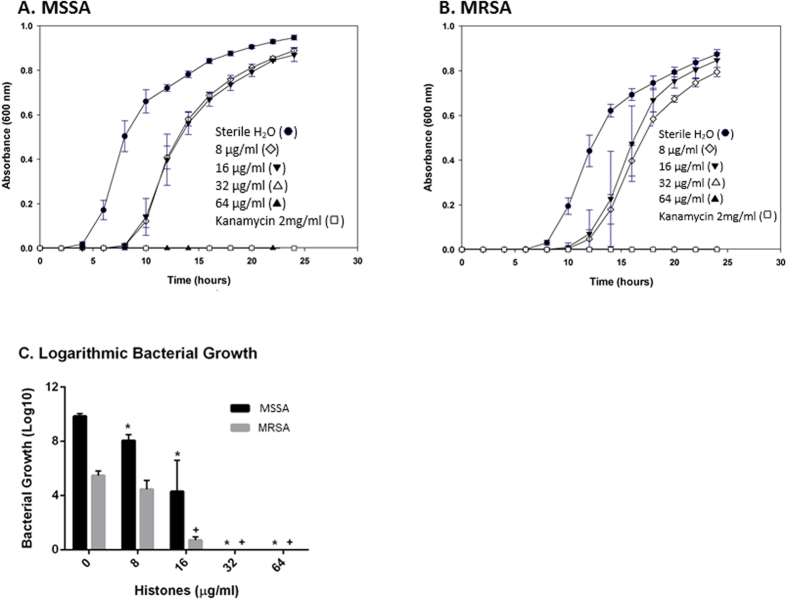
Dose-dependent growth inhibition of biofilms treated with histone mixture (MBEC assay). (**A**) MSSA (**B**) and MRSA strains were treated with increasing concentrations of histone mixture in sterile H_2_O, pH 7.4: 8 μg/ml, 16 μg/ml, 32 μg/ml, 64 μg/ml, and kanamycin 2 mg/ml as a positive control for inhibition. The untreated control cells were incubated with sterile H_2_O, pH 7.4 as a negative control for inhibition. Results for dose-dependent growth inhibition (**C**) are of three independent trials, each in triplicate. Statistical analysis was done by Student’s T-Test, (*) indicates P ≤ 0.002 compared with the untreated control cells for MSSA bacterial growth; (+) indicates P ≤ 0.0001 compared with the untreated control cells for MRSA bacterial growth.

The growth of viable bacteria in biofilms treated with increasing concentrations of histones was monitored using the Minimum Biofilm Eradication Concentration (MBEC) approach. Figure 3A and B represent the growth curves for MSSA and MRSA, respectively. After 24 h of incubation, a significant MSSA growth inhibition was detected at 8 μg/ml of histones (P < 0.002, compared to the untreated control cells), with complete inhibition at 32 μg/ml of histones (Fig. 3A and C). A significant dose-dependent response to the increasing histone concentrations was observed for MSSA growth inhibition only (Fig. 3C). Interestingly, MRSA was more resistant to 8 μg/ml of histones, but only showed a tendency for growth inhibition at this concentration (Fig. 3C). However, as for MSSA, MRSA growth was significantly inhibited at 16 μg/ml of histones and completely inhibited at 32 μg/ml (Fig. 3B and C).

The histone mixture was effective in eradicating both MSSA and MRSA biofilms with MBEC values of 23 ± 5 μg/ml and 21 ± 5 μg/ml, respectively (Fig. 3A and B). While no significant difference was observed between these MBEC values, they are statistically greater (~3–4 fold, P ≤ 0.05) than the MIC (MSSA = 6 ± 1 μg/ml, MRSA = 8 ± 2 μg/ml) and the minimum bactericidal concentration (MBC) (MSSA = 6 ± 1 μg/ml, MRSA = 8 ± 3 μg/ml) values determined for the planktonic bacteria in our previous study^18^.

### Histones affect membrane integrity

Fluorescence microscopic images of both MSSA (Supplementary Fig. 1) and MRSA (Supplementary Fig. 2) biofilms treated with increasing concentrations of histones were acquired (Supplemental file, materials and methods). Low levels of damaged cells innately occur in biofilms; additionally, a gradual increase in red fluorescence was observed at concentrations of 16 μg/ml for the histone mixture and higher for MSSA biofilms (Supplementary Fig. 1, center column). On the other hand, there was no increase in red fluorescence in MRSA biofilms treated with 16 μg/ml of histones, but a substantial increase when treated with 32 μg/ml and higher concentrations (Supplementary Fig. 2, center column).

To quantify the increase in membrane damage, we analyzed the red/green pixel ratio of 36 images for each histone concentration and determined whether the increase in red fluorescence was significantly higher than the untreated controls. An increase in red fluorescence at 16 μg/ml of histones was visually discerned (Supplementary Fig. 1); however, red/green pixel ratios for both MSSA and MRSA were not significant at that concentration (Supplementary Fig. 3). At concentrations of 32 μg/ml of histones and higher, both MSSA and MRSA showed a significant increase in membrane damage compared with untreated control cells (P ≤ 0.003 and P ≤ 0.009 respectively; Supplementary Fig. 3).

### Scanning Electron Microscopy

The effect of histones on bacterial membranes was assessed using scanning electron microscopy (SEM) for MRSA biofilms grown on polycarbonate membrane filters (Supplemental file, materials and methods). Representative SEM images of MRSA biofilms treated with the histone mixture and the negative control are shown in Supplementary Fig. 4. An untreated biofilm population is presented in Supplementary Fig. 4A, where we observed numerous spherical structures embedded in a polymeric matrix consisting of an array of interlacing filaments. A closer view of the untreated MRSA biofilm revealed spherical bacteria (~0.7 μm in diameter) with smooth surfaces (Supplementary Fig. 4B). After treatment with 128 μg/ml of histones, we observed clear morphological differences in the MRSA bacterial population, such as blebbing, collapsing and deformations, indicative of direct damage to bacterial membranes caused by histones (Supplementary Fig. 4C). We identified cells with evidence of indentation of the cellular membrane (Supplementary Fig. 4D), small pore formation (Supplementary Fig. 4C), as well as whole cell collapse (Supplementary Fig. 4E). Similar observations were made for six separate membrane filters of each sample in two separate experiments. No such defects were observed in control populations.

### Gene expression Analysis

**Figure 4:**
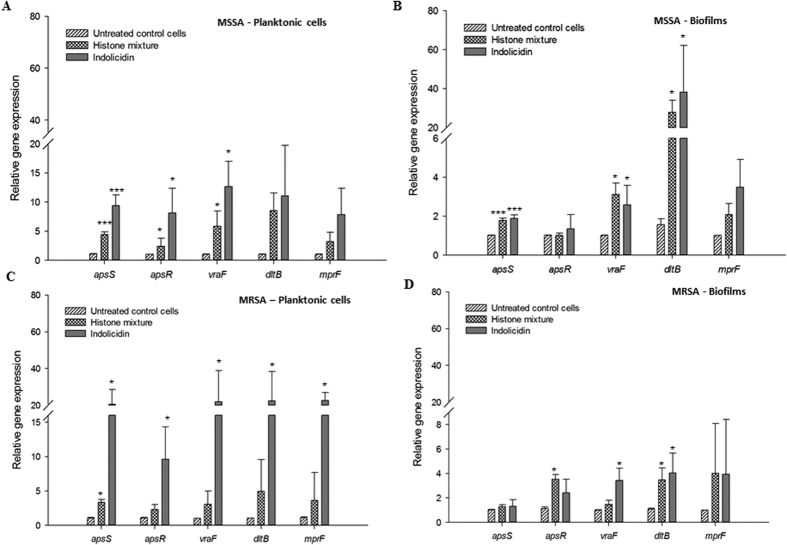
Effect of avian histones on the relative transcripts levels of the genes associated with the AMP resistance mechanisms. (**A**) Planktonic MSSA cells; (**B**) MSSA cells embedded in biofilms; (**C**) Planktonic MRSA cells; (**D**) MRSA cells embedded in biofilms. Values represent mean fold change expression differences compared to untreated control cells ± standard deviation from three independent experiments; *P < 0.05, ***P < 0.001. Calculations of significant differences are versus the corresponding control without addition of CAMP.

The effect of histones on the expression of genes representing AMP resistance mechanisms was determined by quantitative reverse transcription PCR. Sub-MIC incubation in the presence of histones or indolicidin (positive control) increased the transcription of certain resistance genes without affecting the housekeeping genes, *16S rRNA* and *gyrA* (Fig. 4). The relative transcript levels of antimicrobial peptide sensor *apsS*, regulator *apsR* and AMP transporter associated *vraF* genes were increased 4–6 fold (P ≤ 0.05) in MSSA planktonic cells exposed to histones (Fig. 4A). In MRSA planktonic cells, the relative gene expression of *apsS* and *apsR* were increased by 4 and 3 -fold by histones (P ≤ 0.05), respectively (Fig. 4C). The relative expression of *apsS, apsR, vraF, dltB* and *mprF* genes were increased by 2–60 times (P* ≤ *0.05) in MSSA biofilms treated with the histone mixture (Fig. 4B). In MRSA biofilms, the relative expression of *apsR* and *dltB* genes were upregulated by 2–3 times (P* ≤ *0.05; Fig. 4D). The relative expression of the above genes in MSSA and MRSA (biofilms and planktonic cells) were also increased by indolicidin (P ≤ 0.05; Fig. 4).

### Antimicrobial activity of Purified Histones H5

**Figure 5:**
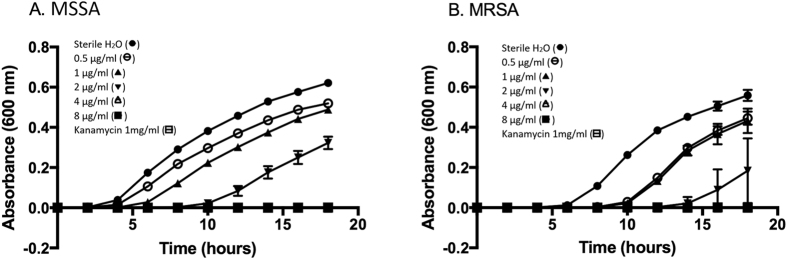
Bacterial growth curves and dose-dependent growth inhibition of bacteria treated with histone H5 (broth microdilution assay). (**A**) MSSA and (**B**) MRSA strains were treated with increasing concentrations of histone H5 in sterile H_2_O, pH 7.4: 0.5 μg/ml, 1 μg/ml, 2 μg/ml, 4 μg/ml, 8 μg/ml, and kanamycin 1 mg/ml as a positive control for inhibition. The untreated control cells were incubated with sterile H_2_O, pH 7.4 as a negative control for inhibition. Results are representative of three independent trials, each in triplicate. Statistical analysis was done by Student’s T-Test, (*) indicates P ≤ 0.01 compared with the untreated control cells for MSSA bacterial growth; (+) indicates P ≤ 0.0001 compared with the untreated control cells for MRSA bacterial growth.

Exposing MSSA and MRSA to increasing H5 concentrations increased the duration of the lag phase which preceded detectable bacterial turbidity. Histone H5 inhibition of each bacterial strain was dose-dependent (Fig. 5A,B). MSSA and MRSA growth was completely inhibited above 4 μg/ml of histone H5. A significant dose-dependent response was observed from 0–8 μg/ml of histone H5 for MSSA (Fig. 5B). The MIC and MBC values of histone H5 versus MSSA and MRSA planktonic bacteria are reported in Table 2. The MIC values of MSSA and MRSA were not significantly different from each other, demonstrating similar susceptibilities to histone H5 (3.8 ± 0.4 μg/ml and 2.4 ± 0.8 μg/ml, respectively).

## Discussion

Biofilm plaques on medical equipment can be traced to 65% of all infections in human medicine^20^. Environmental cues from within an established biofilm can activate dispersal mechanisms, in which some bacteria revert to the planktonic state and are shed from the biofilm to cause acute infections, including sepsis, or form new biofilms at secondary sites leading to chronic infection. Staphylococci, particularly MRSA stains, are a major cause of nosocomial infections due to resistance to numerous current antibiotics^21^, which is exacerbated by biofilm formation^22^. Antibiotics or combinational therapies are commonly used for the treatment of Staphylococcal biofilms. However, multidrug resistant strains, such as MRSA, are reducing the effectiveness of such treatments.

This study documents antimicrobial activity and anti-biofilm activity of histones from chicken erythrocytes against methicillin sensitive and methicillin-resistant *S. aureus.* Biofilm cultures of MSSA and MRSA were equally susceptible to the histone mixture; however, in each case, biofilm cultures displayed 3–4 fold increased resistance compared to their planktonic counterparts. Purified histone H5 enhanced the antimicrobial activity (MIC, MBC) by 3–4 fold against MSSA and MRSA planktonic cells, compared to the histone mixture. Histone H5 had MIC and MBC values of 3.8 ± 0.4 μg/ml and 4.0 ± 0.0 μg/ml against MSSA, respectively, and of 2.4 ± 0.8 μg/ml and 2.6 ± 1.1 μg/ml against MRSA, respectively. Since the MIC and MBC values for each strain were not significantly different from each other (P ≤ 0.05; Table 2), histone H5 exerts potent bactericidal activity towards both *S. aureus* strains used in this study (MBC/MIC ≤ 4)^23^.

Several other CAMPs, including indolicidin, cecropin (1–7), –melittin A (2–9) amide (CAMA) and nisin, have been tested against MRSA biofilms, with MBEC values of 640 μg/ml for all three AMPs, and an MBEC/MIC ratio of 40–80^24^. In another study, human β-defensin 3 (hBD3) against MRSA biofilms demonstrated a reduced colony count after treatment with a 6 x MIC concentration. However, complete biofilm eradication was not observed^25^. Therefore, compared to these CAMPs, the histone mixture retains impressive potency against the biofilm forms of both MSSA and MRSA (MBEC/MIC ratio of 2–3). Based on previously published research, the increase in resistance attributed to the biofilm encapsulated bacteria in our study could be due to diffusional barriers associated with the exopolysaccharide (EPS) matrix^26^. To our knowledge, our study is the first to demonstrate biofilm-eradication properties of antimicrobial histones purified from any tissue or species.

A dynamic pharmacokinetic approach based on *in vitro* kill curves is a logical approach to mimic *in vivo* drug-bacteria interactions^27^. Antimicrobial peptides meet a number of criteria for an ‘ideal’ biocide, specifically the rapid kill rate across a range of microorganisms^28^. In our study, histones exhibited a concentration-dependent fast biocidal activity. However, the time-dependent bactericidal effect was less effective compared to another CAMP, indolicidin. Indolicidin is a naturally occurring small protein found in bovine neutrophils with the highest tryptophan content of any known CAMP. Indolicidin possesses a broad antimicrobial activity against a range of Gram-positive and Gram-negative bacterial strains due to its high affinity for lipopolysaccharides and membrane proteins^29^,^30^. In the present study, the faster kill kinetics of indolicidin compared to the histones are likely attributed to a combination of its smaller size (1.9 vs 16.2 kDa, respectively), and greater hydrophobicity (53% vs 28–45%, respectively)^31^,^32^. The kill kinetics of MSSA and MRSA showed that there are differences in their susceptibility to histones, with MRSA being killed more rapidly. hBD3, a cysteine-rich cationic peptide, has been tested against MSSA and MRSA clinical isolates; MRSA exhibited a stronger resistance compared to MSSA^33^. In another study, persulcatusin, an antimicrobial peptide found in the *Ixodes persulcatus* midgut, had 2–4 fold higher MIC values against MRSA compared to MSSA, suggesting MSSA was more susceptible to this CAMP^34^. In contrast to these reports, our study found equivalent sensitivities to histones (equivalent MICs), but the kill kinetics allowed us to distinguish faster -cidal effects of histones against the tested strain of MRSA compared to MSSA. The equivalent MICs for histones against MSSA and MRSA suggests that mechanisms responsible for methicillin resistance are distinct from those by which histones target bacteria, as previously predicted^8^.

The commonly accepted hypothesis concerning the mechanism of action of histones (through electrostatic interactions) is based on their CAMP-like characteristics^11^. Previously, we have shown that avian histones bind to bacterial cell wall components such as lipopolysaccharide and lipoteichoic acid^18^, indicating that antimicrobial histones interact with the bacterial cell wall. In agreement, we observed surface membrane damage of bacterial cells with histones in both MSSA and MRSA biofilms. The detection of pores and dents on the cell surface of MRSA treated with histones is indicative of bacterial membrane damage. This observation is similar to other CAMPs, such as gramicidin S and peptidyl-glycylleucine-carboxyamide^35^.

**Figure 6:**
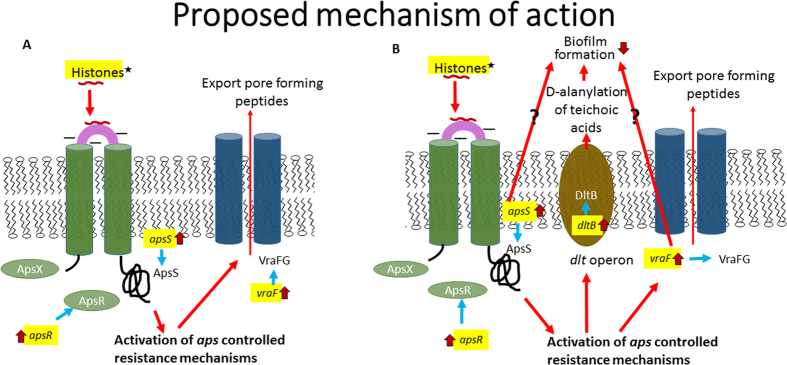
Proposed mechanism of action for the histone sensing system in MSSA and MRSA. (**A**) In planktonic cells, histones bind to the anionic loop of ApsS and trigger the activation of the *apsS* and *apsR* genes encoding the sensor and regulator proteins of *aps* system. This leads to the activation of the *aps* controlled resistance mechanism mediated through *vraFG* transporter genes involved in either exporting the pore forming histones or importing them for proteolytic inactivation. (**B**) In biofilms embedded cells, histone binding to the anionic loop of ApsS leads to the induction of the *dltB* gene encoding *dlt* operon involved in D- alanylation of teichoic acids, in addition to the *vraF* gene involved in peptide transport.

First identified in *Staphylococcus epidermidis*, the antimicrobial peptide sensor (*aps*) system controls the signal transduction and CAMP specific resistance mechanisms in Gram-positive bacteria^11^. CAMPs such as hBD3 induce a gene regulatory response, which is initiated by a high density of negatively charged sensor proteins, ApsS, which detect their specific binding and triggers the *aps* system leading to activation of *aps-* regulated target mechanisms. Other components of *aps-*regulated target mechanisms have been identified in *S. aureus*^36^. Since the constitutive production of the proteins involved in these resistance mechanisms could be a significant metabolic burden to the bacteria, their expression is upregulated only when CAMPs are present^11^. We evaluated upregulation of the *aps* sensor/regulator system in order to obtain mechanistic information concerning the response of MSSA and MRSA to histones, with indolicidin as a positive control. The upregulation of *apsS* with histones indicates there is a similar sensing mechanism in the planktonic cells of both MSSA and MRSA (Fig. 6). The *dltABCD* operon of *S. aureus* plays an important role in the D-alanine activation and synthesis into teichoic acid. *S. aureus* mutants that lack the *dlt* operon are unable to attach to the surface of polyethylene and glass, and consequently are not able to form biofilms^37^. Histones at 0.5 MBEC increased the expression of the *dltB* gene indicating that the *dlt* operon likely functions as a histone inducible sensor by inducing the CAMP resistance mechanisms in the biofilm embedded cells in MSSA, as observed in their planktonic counterparts. Similarly, the biofilm embedded cells in MRSA exhibited significantly increased *aspR, vraF* and *dltB* gene expression upon treatment with the histones. However, in comparison to the bacterial biofilm in MSSA, the fold change in *dltB* gene expression in MRSA was lower. This could be due to the downregulation of basal expression of *dltB* in MRSA.

The histone mixture from chicken erythrocytes possesses both antimicrobial and anti-biofilm properties, which could reflect the synergistic interactions of the histones upon bacterial membranes. Enhanced activity of the histone mixture is relevant since the histones likely exist as an active mixture in the vertebrate immune system, such as in NETs. In contrast to demonstrations of antimicrobial histones isolated from a variety of animal tissues, only nucleated-erythrocytes contain the H5 linker histone^38^. In this report, we have demonstrated for the first time that histone H5 is a powerful antimicrobial agent. Purified histone H5 was equally effective against planktonic MSSA and MRSA and showed enhanced antimicrobial activity compared to the histone mixture (0.63 × MIC of histone mixture vs MSSA and 0.3 × MIC of histone mixture vs MRSA), suggesting that it is a significant contributor to the antimicrobial activity of the mixture in contrast to the other constituents.

### Significance

The current emergence of multi-drug resistant bacteria, such as methicillin-resistant *S. aureus*, is a consequence of the overuse and misuse of antibiotics in medicine and in animal production. The most alarming feature of biofilm-associated infections is their high resistance (10- to 1,000-fold) to conventional antibiotics^39^. This worrisome trend coincides with a significant decline in the discovery and approval of new antibiotics^40^. Consequently, it is estimated that by 2050, approximately 10 million people will be dying per year due to antibiotic-resistant infections^41^. This situation underlines the critical need to develop novel therapeutic agents to treat infectious diseases. Here we show that avian erythrocyte histones and the unique histone H5 are highly effective antimicrobial peptides against the Gram- positive bacterial pathogens MSSA and MRSA. Our data demonstrates, for the first time, that histones are active against the biofilm forms of MSSA and MRSA. This study will aid in the development of alternative antibiotics against antibiotic-resistant bacterial strains and their biofilm forms.

## Materials and Methods

MSSA (ATCC 6538) and MRSA (ATCC 29247) were obtained from the University of Ottawa’s Centre for Research on Environmental Microbiology (Ottawa, ON, Canada). The Kirby Bauer Cefoxitin (FOX 30) disk diffusion test was carried out to demonstrate the antibiotic resistance of the MRSA strain (Supplementary Fig. 5). Unless otherwise specified, Luria-Bertani (LB) agar or broth (BioShop, Canada) was used for the maintenance and growth of bacterial cultures.

### Bacterial strains and growth conditions

Bacterial colonies from glycerol stocks were plated on LB agar (BioShop, Canada) and incubated overnight at 37 °C. Single colonies from LB agar plates were grown in 3 ml of LB broth (BioShop, Canada) overnight at 37 °C, 250 rpm. The inocula were diluted 1:50 in fresh LB broth, and grown until the optical density at 600 nm reached 0.2 (~10^8^ CFUs/ml). For the broth microdilution assay, the bacterial suspension was pelleted at 3000 × g, 4 °C for 10 min, washed with PBS and adjusted to 10^5^ CFUs/ml in PBS. For the MBEC assay and microscopy, the bacterial suspension was adjusted to 10^5^ CFUs/ml in LB broth.

### Acid Extraction of Histones

White Rock chickens were euthanized by decapitation at local slaughterhouses in accordance with Canadian Food Inspection Agency (CFIA) regulations. Blood was collected immediately after decapitation (~37.5 ml of blood/bird with 1.5 mg EDTA/ml). The animals were processed during a normal production cycle of the abattoir, with an agreement in place between the abattoir owners, PI and funding agency. All animal processing procedures were in accordance with CFIA guide-lines and regulations. Erythrocytes were pelleted at 300 × g for 10 min and washed with PBS. Histones were extracted using a protocol developed in our previous study^18^. Briefly, erythrocytes corresponding to 100 ml of blood were lysed with 1000 ml of hypotonic lysis buffer (10 mM Tris-Cl pH 8.0, 1 mM KCl, 1.5 mM MgCl_2_ and 1 mM DTT) for 1 h at 4 °C with light agitation. The extracted nuclei were pelleted at 10,000 × g for 10 min at 4 °C and resuspended in hypotonic lysis buffer. Multiple cycles of pelleting and resuspension were performed in order to remove contaminating soluble proteins. Hemoglobin contamination after each wash was detected in the hypotonic lysis buffer supernatant by a spectral scan (absorbance from 300–500 nm) using the EON microplate spectrophotometer (BioTek, Winooski, VT, USA). When no discernable absorbance between 350 and 450 nm was detected (hemoglobin), the pelleted nuclei were suspended in 400 ml of 0.4 N H_2_SO_4_ for 1 h at 4 °C with light agitation. The nuclear debris was pelleted at 16,000 × g for 10 min at 4 °C and discarded. Histones were precipitated from the supernatant by adding 133 ml of 100% Trichloroacetic acid (Fisher Scientific) in small increments and incubation on ice for 1 h. Histones were pelleted at 16,000 × g for 10 min at 4 °C and washed three times with acetone, without disturbing the pellet, at 16,000 × g for 5 min at 4 °C. The resulting pellet was dissolved in 100 ml of sterile ddH_2_O and passed through an Amicon Ultra centrifugal filter unit (3 kDa MWCO, Millipore Corporation, Billerica, MA, USA) at 750 × g for 30 min. The retained histones were flash frozen using liquid nitrogen and freeze dried overnight (VirTis BenchTop freeze dryer).

### Time Kill kinetic studies

The time kill analyses were performed using a modification of the broth microdilution assay^42^. The histone treatment is based on the minimum inhibitory concentration (MIC) required to prevent any visible increase in bacterial turbidity above baseline (absorbance). Briefly, planktonic cells (50 μl, 10^5^ CFUs/ml) in PBS, pH 7.4 were incubated with histones (50 μl) at different concentrations, ranging from the minimum inhibitory concentration (MIC) of 8 μg/ml to 4 × MIC (32 μg/ml) in a 96-well microplate^18^. Indolicidin (50 μl) (GeneScript, Piscataway, NJ, USA) and sterile water were used as positive and negative controls, respectively. Microplates were incubated for various times (0, 22.5, 45, 90, and 180 min) at 37 °C and 250 rpm, after which 100 μl of LB broth was added to each well. The nominal “time zero” was estimated to be 15 s due to the time required to properly mix/dilute the bacteria and histones followed by the addition of LB broth. The growth of bacteria was measured at 600 nm every 30 min for 16 h at 37 °C with continuous shaking using the EON microplate spectrophotometer and Gen5 data analysis software (BioTek, Winooski, VT, USA). CFU/ml values were calculated from the growth curve lag times using the standard dilution curves that were generated for each trial independently.

### Minimum Biofilm Eradication Concentration (MBEC) Assay

The effect of the histone mixture on biofilm growth was assessed using the MBEC Assay according to the manufacturer instructions (Innovotech Inc., Alberta, Canada). Briefly, wells of a 96-well microplate were inoculated with 150 μl of bacterial inoculum (~10^5^ CFU/ml) and covered with a lid possessing 96 identical pegs. The device was incubated for 24 h at 37 °C and 100 rpm. Following incubation, planktonic cells were rinsed away by immersing the 96-peg lid in a sterile 96-well microplate with 200 μl of sterile PBS. Biofilms were then incubated in the presence of 200 μl of histones that had been dissolved in sterile water, pH 7.4, and serially two-fold diluted in a sterile 96-well microplate. Positive and negative controls for inhibition of growth were assessed by replacing histones with kanamycin (Sigma Aldrich, Oakville, ON) and sterile water, pH 7.4, respectively. The device was incubated for 2 h at 37 °C and 100 rpm, and the 96-peg lid was rinsed twice as described above and placed in a sterile 96-well microplate with 200 μl of LB broth. The device was sonicated for 10 min in order to dislodge the biofilms from the pegs, after which the peg-lid was replaced with a sterile, clear lid. Bacterial growth was monitored using the EON microplate spectrophotometer and Gen5 data analysis software (BioTek, Winooski, VT, USA). The optical density at 600 nm was measured every 30 min over 24 h with continuous shaking (205 rpm) at 37 °C. The lowest histone concentration without visible bacterial growth was designated the minimum biofilm eradication concentration (MBEC). Each microplate contained assigned control wells with serially ten-fold diluted bacterial cultures obtained from the uninhibited biofilm control sample in order to generate standard curves of lag time for bacterial growth versus CFUs/ml. Bacterial growth inhibition at each histone concentration tested (corresponding to the increase in lag time) was determined from this standard curve.

### Quantitative polymerase chain reaction (qPCR) analysis

For gene expression analysis, planktonic MSSA or MRSA with an initial OD_600_ of 0.2 (10^5^ CFU/ml) were cultured with shaking at 160 rpm in LB at 37 °C for 1 h in the presence and absence (untreated control cells) of histones. The corresponding biofilms were formed by incubating the cells (~10^5^ CFU/ml) in a 96-well microplate for 24 ± 2 h with shaking at 160 rpm in LB at 37 °C. Biofilms were then disrupted by repeated washing (3 times) in PBS and the embedded cells were incubated for 1 h in the presence and absence of histones with shaking at 160 rpm. Bacterial cells (planktonic and biofilms) were harvested by centrifugation at 12,000 × g for 10 min. Total RNA was extracted using combination of a Trizol (Invitrogen, Carlsbad, California, USA) and RNeasy Mini kit (Qiagen, Gaithersburg, MD) as described by the manufacturer with some modifications. Briefly, for planktonic cells, lysis was performed by re-suspending the pelleted cells in 20 μl of Proteinase K (10 μg/reaction) and Lysostaphin (200 μg/reaction) (GeneScript, Piscataway, NJ, USA). The mixture was incubated for 15 min at room temperature and total RNA was extracted using Trizol (100 μL/reaction) following the manufacturer instructions. The total RNA was further eluted using RNeasy Mini kit column. For the cells embedded in biofilms, lysis was performed by incubating the pelleted cells for 15 min in a mixture of 40 μl of Proteinase K (20 μg/reaction) and Lysostaphin (400 μg/reaction), followed by total RNA extraction using Trizol and RNeasy Mini kits as described above. The RNA was quantified by NanoDrop ND-2000 spectrophotometer (NanoDrop Technologies Wilmington, DE) and the quality was assessed by agarose gel electrophoresis. RNA from each biological replicate was used for cDNA synthesis using the High Capacity cDNA reverse transcription kit (Applied Biosystems). The relative transcript levels of the genes representing AMP resistance mechanisms: *apsS, apsR, dltB, mprF* and *vraF* were quantified using the Mx3005 P Real time qPCR system (Agilent Technologies, CA, USA). The 20 μl reaction mix contained 4 ng of cDNA, 10 μl Bio-Rad iTaq Universal SYBR green super mix (Bio-Rad North America, Hercules, CA, USA) and 600 nM of gene specific primers (S1 Table). The cationic AMP indolicidin at concentrations of 5 μg/ml (planktonic) or 16 μg/ml (biofilms) was used as positive control. *16S rRNA* was used as an internal housekeeping gene control and its validity was confirmed by verifying concurrence with *gyrB* (another housekeeping gene). The relative expression levels were calculated using the ΔΔCt method.

## Additional Information

**How to cite this article**: Rose-Martel, M. *et al*. Histones from Avian Erythrocytes Exhibit Antibiofilm activity against methicillin-sensitive and methicillin-resistant *Staphylococcus aureus. Sci. Rep.* **7**, 45980; doi: 10.1038/srep45980 (2017).

**Publisher's note:** Springer Nature remains neutral with regard to jurisdictional claims in published maps and institutional affiliations.

## Supplementary Material

Supplemental Files

## Figures and Tables

**Table 1:** Densitometry and LC/MS/MS proteomics analysis results of purified chicken histones.

Bands^a^	Identified proteins^b^	Number of unique peptides^b^	UniProt ID^b^	% of total histone sample (±SD)^c^	Coverage (%)^b^
Band 1	Histone H1.11L	41	P08287	3.7 ± 0.6	49
	Histone H1.01	32	P08284		47
	Histone H1.03	36	P08285		44
Band 2	Histone H1.11R	26	P08288	6 ± 1	51
	Histone H1.01	25	P08284		46
	Histone H1.11L	23	P08287		43
Band 3	Histone H5	25	P02259	18 ± 1	50
Band 4	Histone H3.2	19	P84229	24 ± 1	62
Band 5	Histone H2B 1/2/3/4/6Histone H2B 8Histone H2B 7	27	P0C1H3	19 ± 1	75
		26	Q9PSW9		75
		25	P0C1H5		69
Band 6	Histone H2A (III, IV)	18	P35062, P02263	15 ± 1	72
	Histone H2A.V	6	P02272		54
	Histone H2A (fragment)	3	F1P5G5		16
Band 7	Histone H4	29	P62801	14.6 ± 0.4	73

^a^Bands are depicted in Fig 1. ^b^Data obtained from Scaffold, Proteome Software Inc. ^c^Results obtained from densitometry analysis using ImageJ densitometry software version 1.6; SD, standard deviation.

**Table 2:** MIC and MBC values of purified Histone H5.

Bacteria	Planktonic	
	MIC (μg/ml)^a^	MBC (μg/ml)^b^
**MSSA**	3.8 ± 0.4	4.0 ± 0.0
**MRSA**	2.4 ± 0.8	2.6 ± 1.1

^a^MIC – minimum inhibitory concentration (mean ± SD). ^b^MBC – minimum bactericidal concentration (mean ± SD). Results are representative of three independent trials, each in triplicate. Values represent the mean ± SD of n = 9 replicates at each H5 concentration.
