# Supplementary material for: Histones from Avian Erythrocytes Exhibit Antibiofilm activity against methicillin-sensitive and methicillin-resistant Staphylococcus aureus
Source: Sci Rep. 2017 Apr 5;7:45980. doi: 10.1038/srep45980 (PMC5380990; doi:10.1038/srep45980)
Supplement: Supplemental Files [file srep45980-s1.pdf]

Histones from Avian Erythrocytes Exhibit Antibiofilm activity against methicillin-sensitive and methicillin-resistant *Staphylococcus aureus*.

Megan Rose-Martel[¶1], Garima Kulshreshtha[¶1], Nahom Ahferom Berhane[¶1], Joelle Jodoin[1] and Maxwell T. Hincke[1, 2]*

Affiliations:

[1] Department of Cellular and Molecular Medicine, Faculty of Medicine, University of Ottawa, Ontario, Canada K1H 8M5.

[2] Department of Innovation in Medical Education, Faculty of Medicine, University of Ottawa, Ontario, Canada K1H 8M5.

[¶]These authors contributed equally to this work.

***Corresponding Author:**

Maxwell T. Hincke, Email: mhincke@uottawa.ca

Supplementary Table S1: The primer sequences for qPCR of *S. aureus* gene expression.

Gene	Primer Sequence (5′ → 3′)
*16S rRNA**	TCGTGTCGTGAGATGTTGGG<br>GTTTGTCACCGGCAGTCAAC
*gyrB****	GCGTCCGTTGATTGAAGCG<br>AACGTCACTTGCAACATCGC
*aspS**	CGACGTCTTCAGGTATGGGT<br>ACAGTTGTCCCCTTACCAACA
*aspR**	TGGAACTTGGCGCAGATGAT<br>GCTGTAAACTCATAGACACGACG
*vraF***	CACAAGAAGTGTTGCGAGATATCAAT<br>CCAGATCCAGAGGGACCCATA
*mprF***	AGACGTGCCATTATCTTTGCT<br>TGCTCTACGGAAAGCTACAATAAGC
*dltB***	TGTTTATAGGTTATGGCTATTATGAACGTT<br>TGTAATCACAATGCTAAGTGCTGTTG

Source: *NCBI primer design tool (http://www.ncbi.nlm.nih.gov/tools/primer); ** Li *et al*. (2007) Molecular Microbiology 66: 1136-47; *** Sihto *et al.* (2014) FEMS Microbiology Letters 356: 134-140.

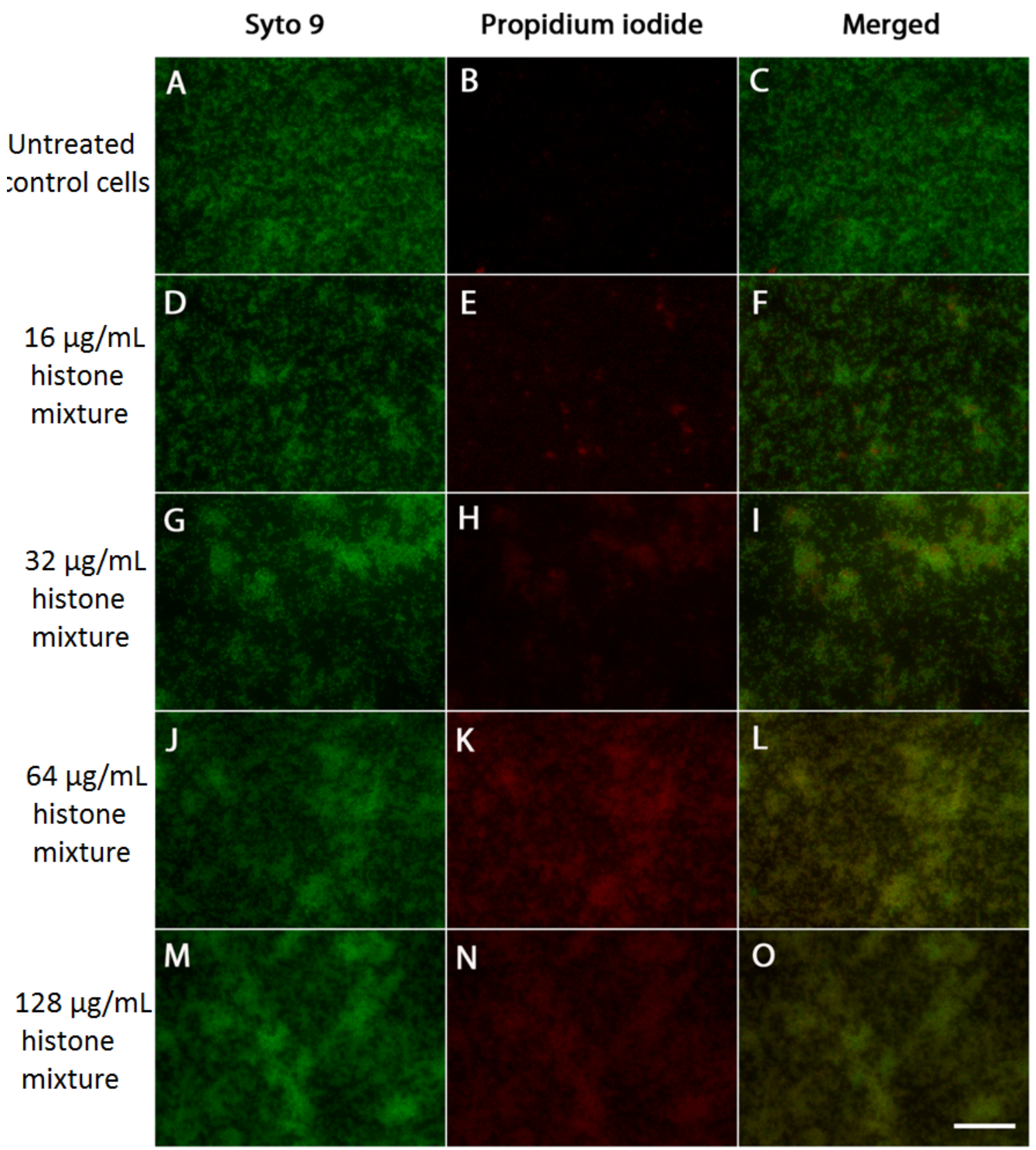

Supplementary Fig 1. Fluorescence microscopy of MSSA biofilms stained for LIVE/DEAD analysis. MSSA biofilms were treated with increasing concentrations of the histone mixture (left) and stained with a combination of Syto-9 (green) and propidium iodide (red) fluorescent dyes. An increase in bacterial permeability is indicated by an increase in red fluorescence (center column) or yellow color in the merged images (right column). Scale bar represents 3 mm. Images are representative of three trials of n = 12.

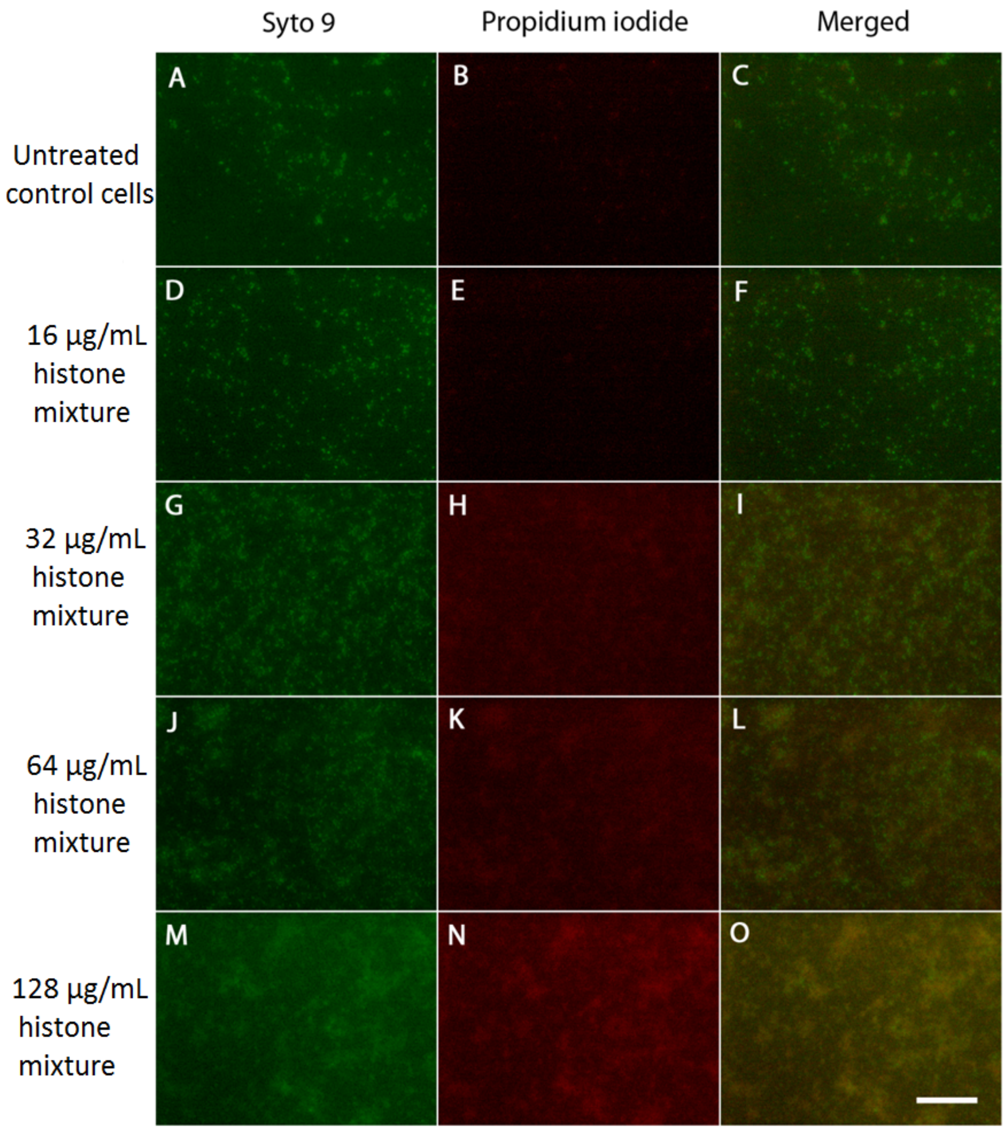

Supplementary Fig 2. Fluorescence microscopy of MRSA biofilms stained for LIVE/DEAD analysis. MRSA biofilms were treated with increasing concentrations of the histone mixture (left)and stained with a combination of Syto-9 (green) and propidium iodide (red) fluorescent dyes. An increase in bacterial permeability is indicated by an increase in red fluorescence (center column) or yellow color in the merged images (right column). Scale bar represents 3 mm. Images are representative of three trials of n = 12.

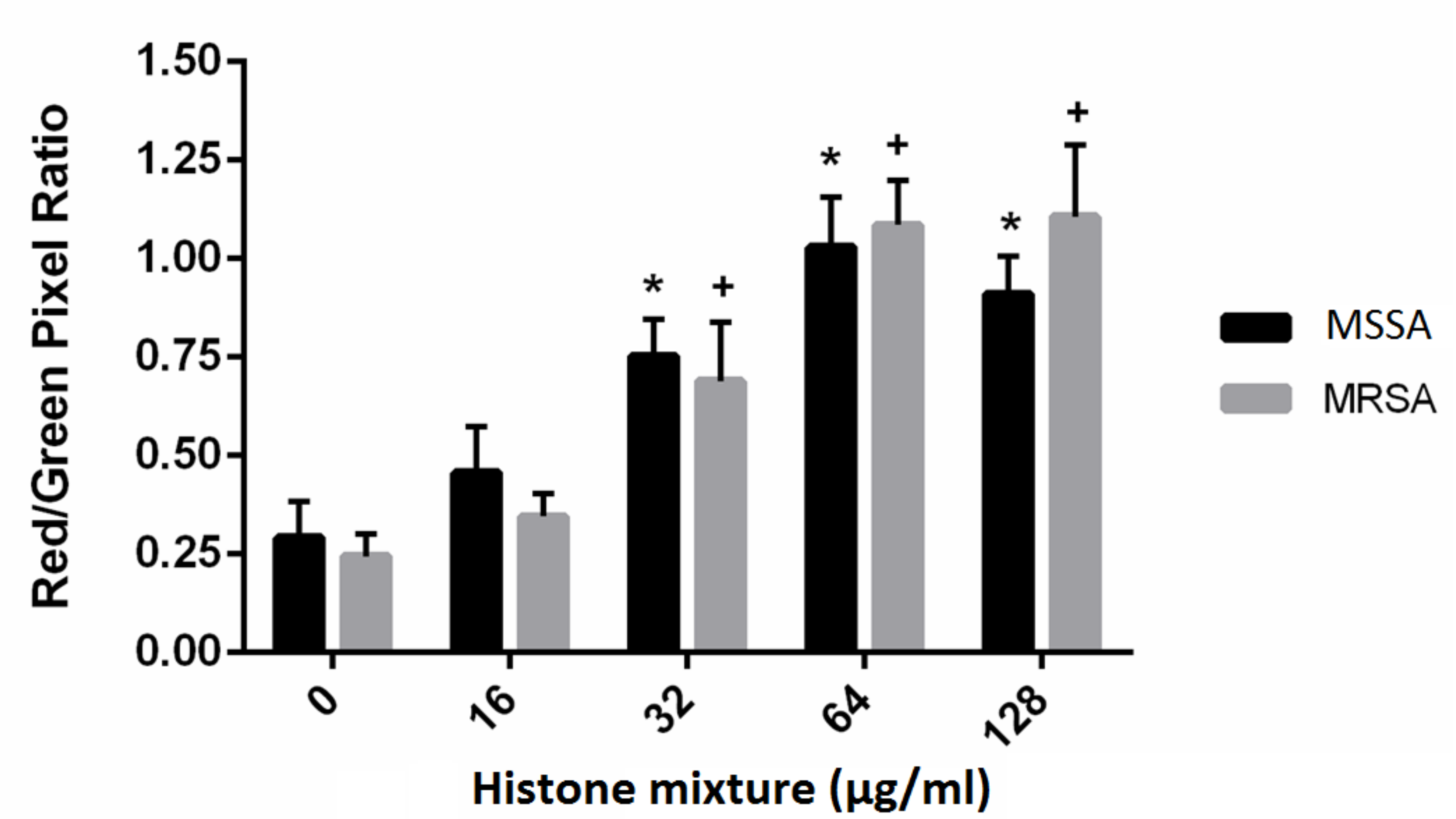

Supplementary Fig 3. Red/Green pixel ratio analysis for fluorescent microscopy images of MSSA and MRSA biofilms. Red and green pixels represent Syto-9 and propidium iodide fluorescence, respectively, following treatment. Statistical analysis was done by Student's T-Test. (*) indicates P ≤ 0.003 compared with the untreated control cells for MSSA; (+) indicates P ≤ 0.009 compared with the untreated control cells for MRSA. Results are of three independent trials of n = 12.

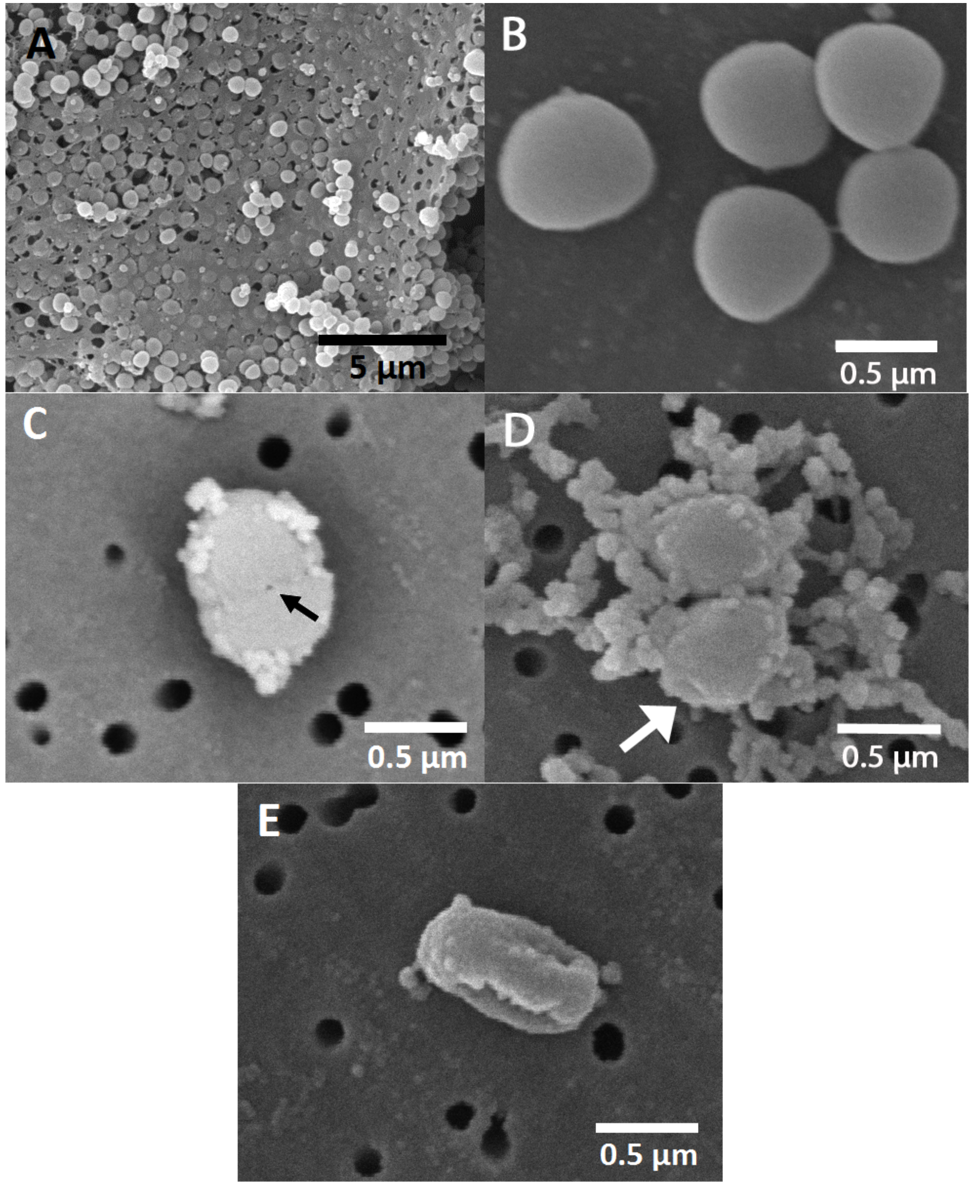

Supplementary Fig 4. Scanning electron microscopy of histone-treated MRSA biofilms. Untreated MRSA biofilms are presented at 5,000 X (A) and 40,000 X magnifications (B). Biofilms treated with 128 µg/ml histones are visualized at 40,000 X (C, D and E), with a white arrow indicating an indentation in the cellular membrane and a black arrow indicating pore formation.

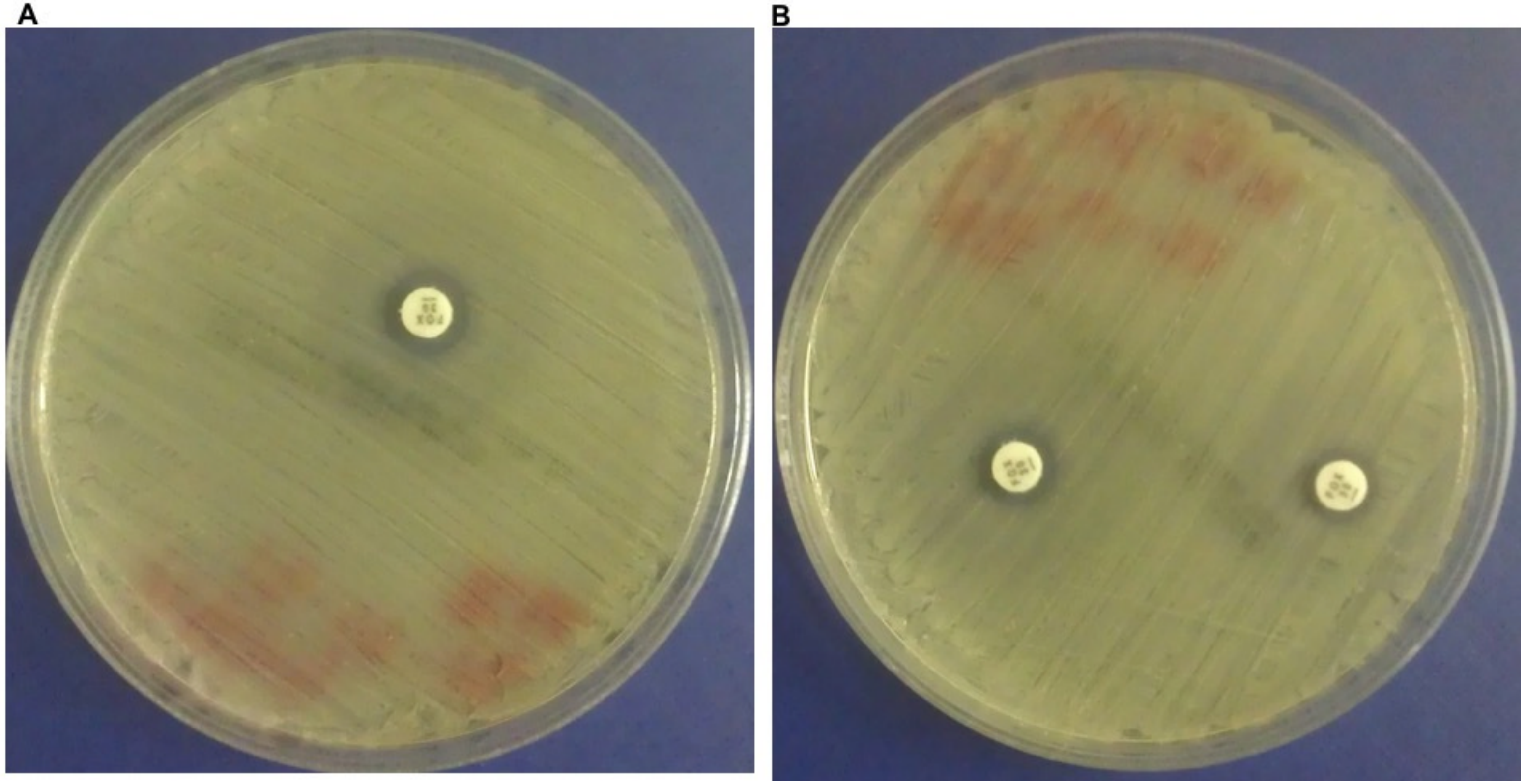

Supplementary Fig 5. The Kirby Bauer Cefoxitin (FOX 30) disk diffusion test on MRSA strain (ATCC 29247). A zone of growth inhibition A. 11mm; B. 10 mm and 9 mm was observed when antibiotic disc containing 30mcg Cefoxitin was tested against the MRSA. Antibiotic disks were acquired from Marc Desjardins – Microbiology Laboratory: The Ottawa Hospital: Cefoxitin (FOX30) Oxoid -30 µg, CT0119B-X3279, Lot #1169215. Plates were incubated for 18 hours at 37 °C. This strain is considered resistant based on the Antimicrobial Interpretation that ≤ 14mm = Resistant (Kern Medical Center Microbiology Lab, Bakersfield College | Kern Community College District | Dr. Janet Fulks, 1801 Panorama Dr. - Bakersfield, CA 93305 - (661)395-4381, http://www2.bc.cc.ca.us/bio16/Lab%20Manual/Lab%20WEb%20Exercises/lab_exercise12.htm)

Materials and Methods

SDS-PAGE and LC/MS/MS Analysis

Samples were analyzed by SDS-PAGE on 15% polyacrylamide gels using the mini-PROTEAN 3 electrophoresis system (Bio-Rad Laboratories, Hercules, CA, USA). Coomassie Blue – stained bands were analyzed by densitometry using ImageJ densitometry software (version 1.6, NIH, Bethesda, MD) and excised for shipping to the Proteomics Platform of the Eastern Quebec Genomics Centre (Laval, QC) for LC/MS/MS proteomics analysis, as previously described [1,2]. Results were analyzed using Scaffold; identification of proteins was deemed valid if two or more peptides were identified at $P \leq 0.05$ probability.

Fluorescence Microscopy

MSSA and MRSA biofilms treated with histones were stained with the LIVE/DEAD® BacLight™ Bacterial Viability Kit (Thermo Fisher Scientific) to assess bacterial viability and membrane integrity. An increase in bacterial membrane damage was detected as an increase in red fluorescence. Wells of a 48-well cell culture plate were inoculated with 250 µl of bacterial inoculum in LB broth (10^5 CFUs/ml) and incubated for 24 h at 37 °C and 100 rpm to allow biofilm formation. Planktonic cells were rinsed away with 300 µl of sterile PBS. Biofilms were then incubated in the presence of 300 µl of histone mixture in sterile water, pH 7.4, for 2 h at 37 °C, 100 rpm. Biofilms were rinsed twice as described above and treated with 300 µl of the LIVE/DEAD® BacLight™ viability stain, containing 6 µM SYTO 9 and 30 µM propidium iodide, for 15 min in the dark. Biofilms were rinsed with 300 µl of sterile PBS to remove any residual dye and visualized using a Leica DMI3000 B fluorescence microscope (Leica

Microsystems, Wetzlar, Germany) and Infinity 3 camera (Lumenera Corporation, Ontario, Canada). The red/green pixel ratio for each image was calculated using Zen 2012 software (Zeiss, Oberkochen, Germany). Images from three trials of n = 12 were analyzed.

Scanning Electron Microscopy

EMD Millipore Isopore polycarbonate membrane filters (0.2 µm pore size, Fisher Scientific, MA, USA) were placed in the wells of a 48-well cell culture plate with 250 µl of bacterial inoculum (10^5 CFU/ml). The plate was incubated for 24 h at 37 °C, after which planktonic cells were removed from the biofilm by rinsing with 300 µl of sterile PBS. Biofilms were treated with 300 µl of histone mixture dissolved in sterile water, pH 7.4, for 2 h at 37 °C and 100 rpm. Biofilms treated equivalently without histones served as a control. Biofilms were rinsed twice with sterile PBS as described above. The filters were fixed with 5% glutaraldehyde in 0.1 M sodium cacodylate, pH 7.5 (VWR, Radnor, PA, USA) overnight at 4 °C. The fixative was removed and the samples were dehydrated using sequential ethanol washes of 20, 40, 60, 80, 90, 95, and twice in 100% for 10 min each. Filters were then treated with 1:2 - hexamethyldisilizane (HMDS):100% ethanol, 2:1 - HMDS:100% ethanol and 100% HMDS for 10 min each (Sigma Aldrich, Oakville, ON, Canada). Filters were air dried overnight, sputter coated with gold to ~10-15nm thickness and viewed using a Tescan Vega-II XMU VPSEM instrument at the Carleton University Nano Imaging Facility (Ottawa, Ontario, Canada).

H5 Purification Using Ion Exchange Chromatography

Linker histones (histones H1 & H5) are selectively soluble in perchloric acid [3]. Therefore, linker histones were extracted as described previously for the histone mixture, except that 10% $HClO_4$ was used instead of 0.4 N H_2SO_4. Histone H5 was then purified from the acid extracted linker histones using a modification of a published protocol [3]. Acid extracted linker histones were dissolved in buffer A (0.35 M NaCl, 10 mM Tris-HCl, pH 8.8), and loaded onto an equilibrated column of CM-Sephadex C-25 resin (GE Healthcare) at a flow rate of 2 ml/min, 4 ˚C. Next, Histone H1 was eluted with elution buffer B (0.75 M NaCl, 10 mM Tris-HCl, pH 8.8), followed by buffer C (1.4 M NaCl, 10 mM Tris-HCl, pH 8.8) to elute histone H5. The histone H5 sample was concentrated (Amicon Ultra centrifugal filter unit, 3 kDa MWCO (Millipore Corporation, Billerica, MA, USA)). Chromatography was repeated three times with the same buffers and resin in order to obtain highly enriched histone H5, as verified by SDS-PAGE. The protein concentration was determined using the BCA protein assay kit (Thermo Fisher Scientific). Purified histone H5 samples (60 µg) in sterile water were aliquoted, lyophilized (VirTis BenchTop freeze dryer) and stored at -20°C. Purity was documented by SDS-PAGE, densitometry and proteomics analysis using Scaffold4 proteome software.

Broth Microdilution Assay

The antimicrobial activity of the extracted histone H5 against planktonic bacterial cells was evaluated using a broth microdilution assay, as modified from a previously described protocol [4]. Purified histone H5 was dissolved in sterile water, pH 7.4, serially two-fold diluted and aliquoted in a 1:1 ratio with bacteria (10^5 CFU/ml) in a 96-well microplate. Kanamycin (Sigma Aldrich, Oakville, ON) and sterile water, pH 7.4 replaced the histones for positive and negative

controls for inhibition of growth, respectively. Microplates were incubated for 3 h at 37 °C and 250 rpm, after which 200 µl of LB broth was added to each well. The EON microplate spectrophotometer and Gen5 data analysis software (BioTek, Winooski, VT, USA) was used to monitor the growth of bacteria by measuring the optical density at 600 nm every 30 min for 18 h with continuous shaking (205 rpm) at 37 °C. The lowest H5 concentration without visible bacterial growth was designated the minimum inhibitory concentration (MIC). Wells containing bacteria at MIC, MIC x 2 and MIC x 4 concentrations of histones were plated on LB agar and incubated at 37 °C for 18 h. The lowest concentration of histones without a single colony was designated the minimum bactericidal concentration (MBC). Each microplate contained wells with serially ten-fold diluted bacterial cultures to generate a standard curve of bacterial lag time versus dilution of initial bacterial culture. Bacterial growth inhibition at each histone concentration was determined from this standard curve.

Statistical Analyses

Student's T-test was used to determine the statistical significance of bacterial growth inhibition between the MIC, MBC and MBEC values of each strain in the broth microdilution assay and MBEC assay. It was also utilized to determine significant variations in bacterial growth when calculating dose-dependent logarithmic inhibition, and to assess differences in bacterial viability when calculating red/green pixel ratio after staining biofilms with the LIVE/DEAD® BacLight™ viability stain. For the time kill kinetic and gene expression analyses, data distribution was tested using the MINITAB 17 (PA, USA). Data showing the normal distribution were compared using ANOVA. Tukey's procedure was used to compare the differences between the least square means. The standard deviation (SD) was reported with the mean values. A P value ≤ 0.05 was necessary for statistical significance.

References

1. Rose-Martel, M., Du, J. & Hincke, M. T. Proteomic analysis provides new insight into the chicken eggshell cuticle. *J Proteomics* **75**, 2697-2706 (2012).

2. Rose-Martel, M., Smiley, S. & Hincke, M. T. Novel identification of matrix proteins involved in calcitic biomineralization. *J Proteomics* **116**, 81-96 (2015).

3. Garcia-Ramirez, M., Leuba, S. H. & Ausio, J. One-step fractionation method for isolating H1 histones from chromatin under nondenaturing conditions. *Protein Express Purif* **1**, 40–4 (1990).

4. Steinberg, D. A. & Lehrer, R. I. Designer assays for antimicrobial peptide. Disputing the "one-size-fits-all" theory. *Methods Mol Biol.* **78,** 169-186 (1997).
